# Health-Related Lifestyle Among College-Going Youth in Bhubaneswar, Odisha

**DOI:** 10.7759/cureus.27208

**Published:** 2022-07-24

**Authors:** Aparajita Mishra, Alpana Mishra, Basanta K Behera, Smruti R Nayak

**Affiliations:** 1 Department of Community Medicine, Kalinga Institute of Medical Sciences, Bhubaneswar, IND; 2 Department of Community Medicine, Kalinga Institute of Medical Sciences, Bhubaneshwar, IND

**Keywords:** youth, lifestyle behavior, junk food, dietary habits, physical activity, lifestyle behaviour

## Abstract

Introduction

Youth is a period where a number of healthy and unhealthy habits get acquired that last throughout a person's life.^ ^Youth health promotion has become a key study focus around the world. Thus this study is undertaken to understand health-related lifestyles affecting college-going youth.

Methods

A cross-sectional survey was done in the junior colleges in Bhubaneswar, India, which included college-going youth. The sample size was 636. The sampling technique was a two-stage stratified sampling method. Firstly, one college was selected from each of five administrative regions (east, west, north, south, and central zone) in Bhubaneswar randomly by lottery method to provide a representative sample. From each college, 128 students were selected randomly from the list of students.

Results

In this study, 237 (37.3%) of the participants ate green leafy vegetables on a daily basis, with 39.3% of females and just 31.2% of males. This difference in preference of green leafy vegetables among males and females was found to be statistically significant with a p-value of 0.019. Out of 180 (28.3%) of the participants who consumed milk products daily, 25% were females, while 38.1% were males (p-value of 0.004). In the present study, out of 247 (38.8%) participants who preferred eating fast food, 37.6% were females, while 42.5% were males. Out of 213 (33.5%) of them who preferred sweets (candy/chocolate), 37.2% were females, while only 22.5% were males. This difference in gender in regards to junk food preference was found to be statistically significant with a p-value of 0.001. In the current study, out of 243 (42%) of the participants who spent 30 minutes to one hour on exercise per day, 43.4% were females, while 37.4% were males. Out of 133 (23%) of them who spent one to two hours on exercise per day, 23.6% were females, while only 20.9% were males (p-value of 0.003). Out of 208 (35.9%) of the participants who preferred walking as the main mode of exercise, 40.2% were females, while 22.3% were males (p-value <0.0001).

Conclusion

The main findings of this study demonstrated that the majority of the youth followed proper meal routines and engaged in regular physical exercise. However, when compared to junk food consumption, participants consumed far fewer green leafy vegetables. This would prove to be harmful to their health. Since the youth of today are the future of tomorrow, they should be provided with proper health education regarding the harmful effects of regular intake of junk food.

## Introduction

Youth is a time when a person develops a variety of healthy and unhealthy habits that will follow them throughout their lives [1-2]. The World Health Organisation (WHO) estimates that nearly two-thirds of premature deaths and one-third of the burden of disease in adulthood are associated with inappropriate behaviors or lifestyles that have begun in the youth. About 27-39% of cancers can be prevented merely by improving dietary habits and physical activity [3-5]. Optimum nutrition, physical activity, and sleep levels have been demonstrated to be important for academic achievement [6-8]. There is strong evidence that the main health behaviors associated with health status and quality of life for the youth are daily consumption of fruits and vegetables, physical activity, avoidance of sedentary behavior, and abstinence from alcohol and tobacco [9-10].

Physical activity is a key determinant of energy expenditure and thereby fundamental to energy balance and weight control [11]. Participation in physical activity is also important for ensuring sound mental health [12]. Intense and frequent physical activity is seen to be associated with lower levels of tension and fatigue in the youth [13]. Studies have shown that there is a positive association between high levels of physical activity, consumption of healthy foods with good health, and health-related quality of life [14-15]. Health-promoting lifestyle among the youth has become a major research focus globally. Life of college-going students is a transitional period; hence they can be offered good opportunities for establishing health-promoting lifestyles. This study aims to assess the lifestyle domains of the youth in terms of dietary habits and physical activity and to find out the risk factors associated with lifestyle domains.

## Materials and methods

A cross-sectional survey was done in the junior colleges in Bhubaneswar, India. The total period of study was from October 2019 to September 2021. The study participants included college-going youth.

The sample size was 636. The sampling technique was a two-stage stratified sampling method. Firstly, one college was selected from each of five administrative regions (east, west, north, south, and central zone) in Bhubaneswar randomly by lottery method to provide a representative sample. From each college, 128 students were randomly selected from the list of students.

College-going youth who gave written informed consent were included in the study. The students who were sick with physical disabilities and were absent on the day of data collection were excluded.

Permission to conduct the study was obtained from the Institutional Ethics Committee (IEC) of Kalinga Institute of Medical Sciences (KIMS), with approval ref no: KIMS/KIIT/IEC/87/2019 dated 06.09.2019, before the commencement of the study.

A pre-designed, pre-tested, semi-structured questionnaire was prepared based on previous similar research articles in the Department of Community Medicine, KIMS, and was used to collect the socio-demographic data and relevant variables using the UNICEF findings of the National Research on Adolescents Attitude to Healthy Lifestyle, and Skills Questionnaire (2018) [16] and the step-wise approach to "Noncommunicable disease risk factory survey", questionnaire by WHO (2009) [17]. The questionnaire was divided into three sections:

1) socio-demographic profile (modified Kuppuswamy 2019 scale was used to assess the socio-economic status [18]);

2) dietary habits (e.g., breakfast habits, number of meals per day, frequency of consumption of green leafy vegetables, preference for junk foods, etc.);

3) physical activity habits.

The data was entered in Microsoft Excel 2019 (Microsoft® Corp., Redmond, USA), and data cleaning was done to eliminate typographical errors and harmonize the data set. SPSS version 20.0 (IBM, Inc., Armonk, USA) was used for analysis. 

## Results

In the current study, a total of 636 participants (476 females and 160 males) were selected randomly from five selected colleges (chosen randomly from each of the five administrative regions of Bhubaneswar Municipal Corporation according to pre-determined inclusion and exclusion criteria).

Out of 636 students, the majority of 83.1% were in the age group of 15-17 years with a mean age of 17.6±1.22 years. The maximum age was 23 years and the minimum age was 15 years.

In the current study, the majority (74.8%) of the participants were females and 25.2% were males. There were 56.4% of them from a nuclear family, followed by 37.6% from a joint family. In the current study, the maximum number (60.5%) of the participants belonged to the upper-middle socio-economic class according to the modified Kuppuswamy socio-economic scale [18] (Table 1).

**Table 1:** Demographic characteristics of the study participants (n=636)

Variable	Number (%)
Age (in years)	
15 - 17	528 (83.1)
18 - 20	88 (13.8)
21 - 23	20 (3.1)
Sex	
Male	160 (25.2)
Female	476 (74.8)
Type of family	
Nuclear	359 (56.4)
Joint	239 (37.6)
Three generation	38 (6)
Residency	
Rural	158 (24.8)
Urban	478 (75.2)
Staying at	
Home	596 (93.7)
Hostel	40 (6.3)
Religion	
Hindu	617 (97.0)
Muslim	13 (2.1)
Christian	6 (0.9)

In this study, out of 424 (66.7%) participants who always used to have breakfast regularly, 68.8% were males and 66.0% were females. Out of 349 (54.9%) of the participants who had >2 meals/day, 54.4% were females, while 56.2% were males. Here 287 (45.1%) of them had <2 meals/day, of which 45.6% were females and 43.8% were males, but the difference observed was not statistically significant (Table 2).

**Table 2:** Gender distribution of study participants according to dietary habits (n=636)

	Male, n (%)	Female, n (%)	Total number (%)
Eat breakfast every day			
Always	110 (68.8)	314 (66.0)	424 (66.7)
Sometimes	35 (21.9)	122 (25.6)	157 (24.7)
Rarely	10 (6.2)	30 (6.3)	40 (6.3)
Never	5 (3.1)	10 (2.1)	15 (2.3)
Chi-square=1.35, df=3, p=0.716			
Eat regularly at right time every day			
Always	65 (40.6)	191 (40.1)	256 (40.3)
Sometimes	63 (39.4)	217 (45.6)	280 (44.0)
Rarely	12 (7.5)	42 (8.8)	54 (8.5)
Never	20 (12.5)	26 (5.5)	46 (7.2)
Chi-square=9.5, df=3, p=0.023			
Drink tea/coffee			
Never	49 (30.6)	183 (38.4)	232 (36.4)
Occasionally	59 (36.9)	201 (42.2)	260 (40.9)
1 - 2 times/day	44 (27.5)	85 (17.9)	129 (20.3)
3 - 4 times/day	6 (3.8)	6 (1.3)	12 (1.9)
>4 times/day	2 (1.2)	1 (0.2)	3 (0.5)
Chi-square=15.01, df =4, p=0.005			
Number of meals/day			
<2 meal	70 (43.8)	217 (45.6)	287 (45.1)
>2 meal	90 (56.2)	259 (54.4)	349 (54.9)
Chi-square=0.16, df=1, p=0.37			

Out of 184 (28.9%) of the participants who were not having a non-vegetarian diet, 33.4% were females, while only 15.6% were males. This difference in the food preference among males and females was found to be statistically significant with a p-value of 0.002. 

In this study, 237 (37.3%) of the participants were eating green leafy vegetables daily of which 39.3% were females, while 31.2% were males. This difference in preference of green leafy vegetables among male and female adolescents was found to be statistically significant with a p-value of 0.019.

Out of 201 (31.6%) of the total participants who had milk products more than two times per week, 33.1% were males, while 31.1% were females. Out of 180 (28.3%) of the participants who had milk products daily, 25% were females, while 38.1% were males. This difference in preference of milk products between male and female participants was statistically significant with a p-value of 0.004 (Table 3).

**Table 3:** Gender distribution of study participants according to the frequency of food consumption (n=636)

	Male, n (%)	Female, n (%)	Total number (%)
Frequency of eating non-vegetarian diet			
Not eating	25 (15.6)	159 (33.4)	184 (28.9)
< once/week	23 (14.4)	88 (18.5)	111 (17.5)
2 - 4 times/week	80 (50.0)	177 (37.2)	257 (40.4)
>4 times/week	32 (20.0)	52 (10.9)	84 (13.2)
Chi-square=26.5, df=3, p=0.002			
Frequency of eating green leafy vegetables			
Daily	50 (31.2)	187 (39.3)	237 (37.3)
4 - 6 times/week	44 (27.5)	83 (17.4)	127 (20.0)
2 - 3 times/week	52 (32.5)	141 (29.6)	193 (30.3)
0 - 1 time/week	10 (6.2)	36 (7.6)	46 (7.2)
Not eating	4 (2.5)	29 (6.1)	33 (5.2)
Chi-square=11.73, df=4, p=0.019			
Frequency of eating whole fruit			
Daily	21 (13.1)	78 (16.4)	99 (15.6)
4 - 6 times/week	31 (19.4)	92 (19.3)	123 (19.3)
2 - 3 times/week	64 (40.0)	172 (36.1)	236 (37.1)
0 - 1 time/week	31 (19.4)	86 (18.1)	117 (18.4)
Not eating	13 (8.1)	48 (10.1)	61 (9.6)
Chi-square=1.89, df=4, p=0.756			
Frequency of eating milk products			
Daily	61 (38.1)	119 (25.0)	180 (28.3)
4 - 6 times/week	20 (12.5)	46 (9.7)	66 (10.4)
2 - 3 times/week	33 (20.6)	102 (21.4)	135 (21.2)
0 - 1 time/week	24 (15.0)	102 (21.4)	126 (19.8)
Not eating	22 (13.8)	107 (22.5)	129 (20.3)
Chi-square=15.25, df=4, p=0.004			

**Figure 1:**
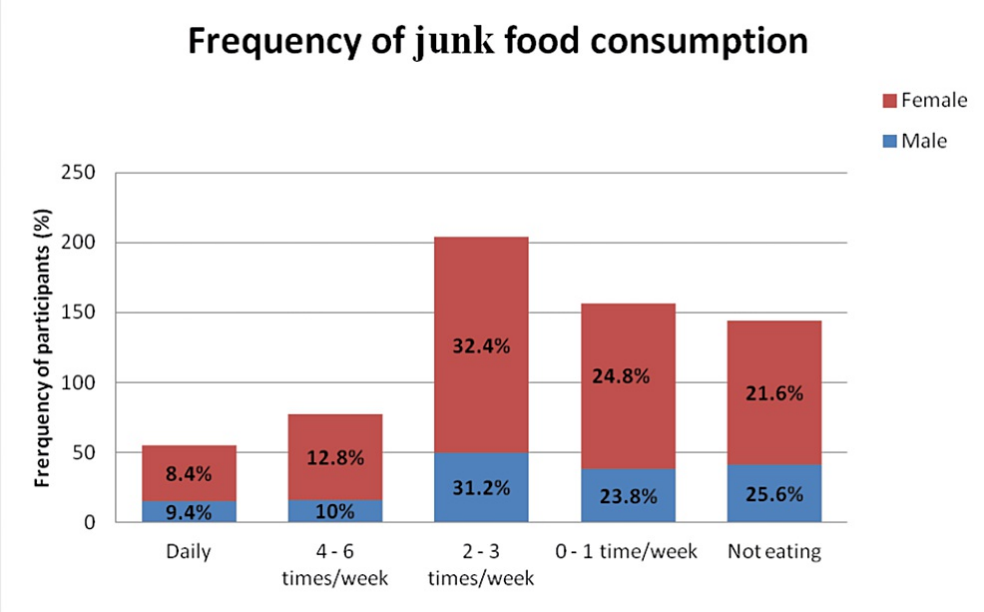
Distribution of study participants according to the frequency of junk food consumption (n=636)

Out of 281 (44.2%) of the participants who preferred junk foods (foods high in energy, low in nutrient content and/or high in fat) more than two times/week, 45.2% were females, while 41.2% were males. This difference was not statistically significant with a p-value of 0.763 (Figure 1).

**Figure 2:**
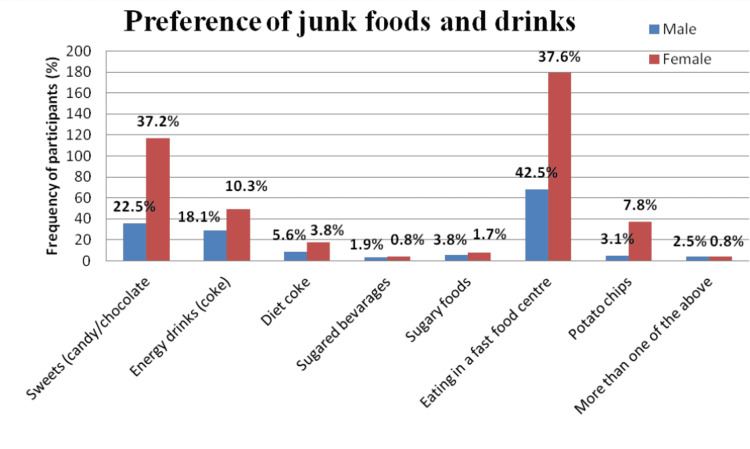
Distribution of study participants according to their preference of junk foods and drinks (n=636)

In the present study, out of 247 (38.8%) participants who preferred eating fast food, 37.6% were females, while 42.5% were males. Out of 213 (33.5%) of them who preferred sweets (candy/chocolate), 37.2% were females, while only 22.5% were males. This difference in gender in regard to junk food preference was found to be statistically significant with a p-value of 0.001 (Figure 2).

In the current study, out of 243 (42%) of the participants who spent 30 minutes to one hour on exercise per day, 43.4% were females, while 37.4% were males. Out of 133 (23%) of them who spent one to two hours on exercise per day, 23.6% were females, while 20.9% were males. This gender difference was found to be statistically significant with a p-value of 0.003. Out of 208 (35.9%) of the participants who preferred walking as the main mode of exercise, 40.2% were females, while 22.3% were males. This difference was found to be statistically significant with a p-value of <0.0001.

In the current study, out of 309 (48.6%) of the participants who played badminton, 56.1% were females, while 26.2% were males. Out of 151 (23.7%) of those who did not play any of the following outdoor games, 25.4% were females, while 18.8% were males. This difference in their playing habits was found to be statistically significant with a p-value of <0.0001 (Table 4). 

**Table 4:** Gender distribution of participants according to their physical activity habits (n=636)

	Male, n (%)	Female, n (%)	Total number (%)
Following exercise routine			
Never	21 (13.1)	36 (7.6)	57 (9.0)
Occasionally	59 (36.9)	207 (43.5)	266 (41.8)
Always	80 (50.0)	233 (48.9)	313 (49.2)
Chi-square=5.412, df=2, p=0.067			
Time spent on exercise per day among those who exercise (n=579)			
<30 min	26 (18.7)	98 (22.3)	124 (21.4)
30 min - 1 hour	52 (37.4)	191 (43.4)	243 (42)
1 hour - 2 hour	29 (20.9)	104 (23.6)	133 (23)
>2 hour	32 (23.0)	47 (10.7)	79 (13.6)
Chi-square=13.67, df=3, p=0.003			
Mode of exercise among those who regularly exercise (n=579)			
Walking	31 (22.3)	177 (40.2)	208 (35.9)
Running	52 (37.4)	78 (17.7)	130 (22.5)
Weight lifting	18 (12.9)	29 (6.6)	47 (8.1)
Dancing	2 (1.4)	105 (23.9)	107 (18.5)
Others: swimming/cycling	19 (13.7)	21 (4.8)	40 (6.9)
More than one of the above	17 (12.2)	30 (6.8)	47 (8.1)
Chi-square=77.59, df=5, p<0.0001			
Playing outdoor games (n=636)			
No	30 (18.8)	121 (25.4)	151 (23.7)
Football	23 (14.4)	17 (3.6)	40 (6.3)
Basketball	6 (3.8)	18 (3.8)	24 (3.8)
Table tennis	0 (0.0)	6 (1.3)	6 (0.9)
Lawn tennis	0 (0.0)	1 (0.2)	1 (0.2)
Badminton	42 (26.2)	267 (56.1)	309 (48.6)
Other	51 (31.9)	26 (5.5)	77 (12.1)
More than one of the above sports	8 (5)	20 (4.2)	28 (4.4)
Chi-square=117.94, df=7, p<0.0001			

## Discussion

In the current study, out of 636 students, the majority (83.1%) were in the age group of 15-17 years. Most of the participants belonged to the early adolescent age group. The mean age of the participants was 17.6±1.22 years. In the current study, the majority (74.8%) of the participants were females, and 25.2% were males. These findings were in contrast to the study conducted by Elsabagh et al. [4], where 66% of the participants were aged between 19-21 years, and 34% were less than 19 years of age, having a mean age of 19.26±1.17 years. 

In the current study, the maximum number (60.5%) of participants belonged to the upper-middle socio-economic class, while in a study by Kulkarni [19], 70% of the youth belonged to upper socio-economic status as per modified Prashad's classification, which was nearly similar to the findings of our study.

In the current study, out of 66.7% of participants who always used to have breakfast regularly, 68.8% were males, and 66.0% were females. This finding shows that an almost equal proportion of females and males were used to consume breakfast always. This finding was inconsistent with the study by Musaiger et al. [20],** **where 56.7% of the males consumed breakfast regularly compared to 47.6% of females. Similarly, in a study by Kulkarni [19], the habit of skipping breakfast was more common among females (37.09%) as compared to males (24.14%). 

In the current study, out of 54.9% of the participants who had >2 meals/day, 54.4% were females, while 56.2% were males. Here 45.1% of them had <2meals/day, of which 45.6% were females, and only 43.8% were males; whereas, in a study by Ganesan et al. [1], 94.7% of the females had more than two meals/day while 90.6% of the males were having less than two meals/day, which was contrary to the findings of our study.

In the current study, 37.3% of the participants were eating green leafy vegetables daily, of which 39.3% were females, while only 31.2% were males. Out of 50.3% of the participants who had green leafy vegetables more than two times/week, 60% were males, while 47% were females. Likewise, in a study by Musaiger​​​​​​​ et al. [20], 51.9% of the females were consuming vegetables more than four times per week, compared to 40.4% of the male participants. In a study by Ganesan et al. [1], 36.2% of male participants had green leafy vegetables in comparison to 33.5% of the female participants, which disagreed with the findings of our study, where more number of females were seen to be consuming green leafy vegetables. 

Out of 31.6% of the total participants who had milk products more than two times per week, 33.1% were males, while 31.1% were females. Out of 28.3% of the participants who had milk products daily, 25% were females, while 38.1% were males (p-value of 0.004). This difference is mostly due to cultural influences; whereas, in a study by Musaiger​​​​​​​ et al. [20], 75.3% of the male participants were seen to be taking milk products more than four times per week compared to 58.3% of the female participants. This finding was in contrast with the findings of our study.

In the present study, more males preferred eating fast food as compared to females. In a study by Musaiger​​​​​​​ et al. [20], more females (90.4%) were seen to consume fast foods as compared to 80.9% of males. Similarly, in a study by Kulkarni [19], 46.5% of the males were seen to consume fast foods more than five times per week as compared to females (37.1%). More junk food consumption by males could be attributed to more frequent pocket money obtained by the males. In a study by Ganesan et al. [1], 64% of females preferred eating fast food compared to 54.8% of males. This finding was in contrast with the findings of our study. Out of 33.5% of them who preferred sweets (candy/chocolate), 37.2% were females, while only 22.5% were males. Similarly, in a study by Musaiger​​​​​​​ et al. [20], 85% of the females preferred canned sugared beverages as compared to 70.2% of males, and 65.2% of the females preferred sweets as compared to 64% of males. Hence, this finding was also similar to the findings of our study, where more females had a greater preference for a variety of junk foods. 

In the current study, out of 42% of the participants who spent 30 minutes to one hour on exercise per day, 43.4% were females, while 37.4% were males (p-value of 0.003). Out of 23% of them who spent one to two hours on exercise per day, 23.6% were females, while 20.9% were males. In a study by Evangeline et al. [21], 97.6% of the males were seen to be spending more time on exercise compared to 2.4% of females, which was in contrast with the findings of our study.

Out of 35.9% of the participants who preferred walking as the main mode of exercise, 40.2% were females, while only 22.3% were males; whereas, in a study by Evangeline et al. [21], 97.6% of the males were seen to prefer walking as the main mode of exercise as compared to 2.4% of the females, which was in contrast with the findings of our study.

Out of 23.7% of them who did not play any of the following outdoor games, 25.4% were females, while 18.8% were males; whereas, in a study byEvangeline et al. [21], 97.6% of the males were engaged in outdoor games compared to 2.4% of the females which was in contrast to the findings of our study. In the current study, females are less engaged in outdoor games. This can be attributed to the fact that females are more conservative and are involved in household activities to a greater extent as compared to males. 

Our study has a few limitations. Firstly, the instrument used to assess the health-related lifestyle and health risk behaviors was a self-administered questionnaire; thereby, social desirability bias might have occurred. Secondly, as this study was conducted in a teaching institution, it might not be representative of the whole community. Youth who dropped out of school missed the participation in the study. Finally, being a cross-sectional study design, it limits the temporal association of the risk factors to lifestyle behaviors.

## Conclusions

The main findings of this study demonstrated that the majority of the youth followed proper meal routines and engaged in regular physical exercise. However, when compared to junk food consumption, participants consumed far fewer green leafy vegetables. This would prove to be harmful to their health. A healthy lifestyle is very important to promote good health. Since youth plays an important role in developing and forming healthy lifestyles and behaviors, it is very important that healthy behaviors are promoted from an early age.

After having an understanding of the findings of the study, an improvement in green leafy vegetable consumption is recommended for the youth. Since there was increased consumption of junk foods by the youth, most probably due to the easily available fast food centers located in the vicinity of the college campus, hence the selling of fast foods should be prohibited inside the college campus and should be replaced by various healthier tasty foods in the college canteen. This needs to be done in order to promote healthy eating habits. Along with the teaching institute, this study could be conducted at the community level for a better assessment of the lifestyle habits of non-college-going youth.
